# Supplementary material for: Predictive factors of relapse after dose reduction of oral 5-aminosalicylic acid in patients with ulcerative colitis in the remission phase
Source: PLoS One. 2021 Aug 4;16(8):e0255620. doi: 10.1371/journal.pone.0255620 (PMC8336875; doi:10.1371/journal.pone.0255620)
Supplement: Attachment — Submitted filename: Response to Reviewers_Madarame.docx [file pone.0255620.s002.docx]

June 20, 2021

**Dr. Emily Chenette**

Deputy Editor-in-Chief, *PLOS ONE*

PONE-D-21-04470

Title: Predictive factors of relapse after dose reduction of oral 5-aminosalicylic acid in patients with ulcerative colitis in the remission phase

Dear Editor and Reviewers,

I would like to respond to the points you made.

**Journal Requirements**

1. Support information has been added in accordance with PLOS ONE's style requirements.
2. It was noted that the IRB has waived the requirement for informed consent.
3. In my cover letter, I stated "The authors received no specific funding for this work".
4. Careful corrections have been made regarding excerpts from previous publications.

**Reviewer #1**

1. As you said, I wish I could have shown endoscopic Mayo subscore. However, there were 29 patients who received colonoscopy within 3 months prior to dose reduction of oral 5-ASA and 48 patients within 1 year. This study demonstrated the clinical background that should be carefully considered when reducing the dose of 5-ASA, apart from endoscopic Mayo subscore.

2. I think you are right. I've added it to the text.

2-2. In Japan, fecal calprotectin has been available for measurement since June 2017. Therefore, it was almost impossible to measure fecal calprotectin in this study.

3. It is presented in the text. Also, the detailed the cumulative dose of corticosteroids was added to the S1 Appendix.

4. Instead of recommending a 5-ASA dose reduction for people without a history of ASUC, we have changed the recommendation to be more cautious about 5-ASA dose reduction for patients with a history of ASUC.

**Reviewer #2:**

Patients had to be in remission without combination therapy (steroids or other drugs) for at least 3 months prior to 5-ASA dose reduction. Therefore, none of them were using steroids in combination at the time of reducing the dose of 5-ASA. The exclusion criteria in the text have been clarified.

Figures: I have added Y-axes labels.　We used PACE to improve the quality and appearance of the figures.

Typo: I have corrected "traial" to "trial".

Yours sincerely,

**Akira Madarame, MD**

Department of Gastroenterology and Hepatology, Tokyo Medical University Hospital. 6-7-1 Nishishinjuku, Shinjuku-ku, Tokyo 160-0023, Japan. Tel.: +81-(0)3-3342-6111 (ext. 5913). Fax: +81-(0)3-5381-6654. E-mail: madarame@tokyo-med.ac.jp
